# Catalpol Induces Neuroprotection and Prevents Memory Dysfunction through the Cholinergic System and BDNF

**DOI:** 10.1155/2013/134852

**Published:** 2013-09-08

**Authors:** Dong Wan, LiJun Xue, HuiFeng Zhu, Yong Luo

**Affiliations:** ^1^Department of Emergency, The First Affiliated Hospital of Chongqing Medical University, Chongqing 400016, China; ^2^Chongqing Chemical Industry Vocational College, 400020, China; ^3^College of Pharmaceutical Sciences and Traditional Chinese Medicine, Southwest University, Chongqing 400715, China; ^4^Chongqing Engineering Research Center for Pharmacological Evaluation, Chongqing 400715, China; ^5^Department of Neurology, The First Affiliated Hospital of Chongqing Medical University, Chongqing 400016, China

## Abstract

To investigate the role and mechanism of catalpol on neuroprotective effects and memory enhancing effects simultaneously, neuroprotective effects of catalpol were assessed by neurological deficits score, TTC staining, and cerebral blood flow detecting. Morris water maze was employed to investigate its effects on learning and memory and then clarify its possible mechanisms relating the central cholinergic system and BDNF. Edaravone and oxiracetam were used for positive control drugs based on its different action. Results showed that catalpol and edaravone significantly facilitated neurological function recovery, reduced infarction volume, and increased cerebral blood flow in stroke mice. Catalpol and oxiracetam decreased the escape latency significantly and increased the numbers of crossing platform obviously. The levels of ACh, ChAT, and BDNF in catalpol group were increased in a dose-dependent manner, and AChE declined with a U-shaped dose-response curve. Moreover, the levels of muscarinic AChR subtypes M_1_ and M_2_ in hippocampus were considerably raised by catalpol. These results demonstrated that catalpol may be useful for neuroprotection and memory enhancement, and the mechanism may be related to the central cholinergic system.

## 1. Introduction

Ischemic stroke remains a leading cause of mortality and long-term disability worldwide in adults. Tissue plasminogen activator (tPA) is the only approved drug for acute ischemic stroke, approved by the Food and Drug Administration for ischemic stroke treatment since 1996 [1]. However, only 1-2% of patients can receive thrombolytic therapy mainly due to the short time window (no more than 3–6 h after stroke) and the possible complication such as hemorrhagic transformation (HT) and brain edema [2]. Although edaravone, a free radical scavenger [3, 4], has been used in Asian countries for many years, it is still not approved by the United States and Europe because of its side effects in liver and kidney and only a small clinical trial found that edaravone might improve outcomes [5–7]. Thus, additional effective new drugs are urgently needed. 

Cognitive impairment is a common consequence of stroke affecting one to three quarters of the patients [8, 9], slowing down physical functional recovery [10, 11]. Therefore, targeting cognitive impairments could be a viable approach to facilitate the physical and mental functional recoveries [12, 13]. To date, oxiracetam is shown to improve cognitive ability through increasing brain ACh concentration [14], but its neuroprotective effects on stroke are still not known. In fact, there is a general lack of effective drugs in clinic against cerebral ischemic damage and cognitive deficits simultaneously. So it becomes very important to find out a new drug to treat stroke and improve their cognitive impairment.

Cholinergic signaling is involved in central cognitive processes such as learning and memory [15, 16]; cholinergic deficit is a major neuropathological feature that is associated with memory loss and closely correlated with the severity of cognitive dysfunction in AD [16] and poststroke cognitive impairments [17, 18]. Cholinergic transmission is terminated mainly by ACh hydrolysis through the acetylcholinesterase (AChE) which is responsible for degradation of ACh to acetate and choline in the synaptic cleft [19]. Thus, modulation of the cholinergic signaling pathway, such as inhibition of AChE, activation of ChAT, and promotion of ACh synthesis, may serve as strategies for the treatment of memory dysfunction due to AD [20] or poststroke cognitive impairments [17, 18]. 

Catalpol is an important iridoid glycosides compound purified from *Rehmannia glutinosa Libosch* which has been widely used as a traditional Chinese herbal medicine for the treatment of aging diseases and stroke. Our previous research revealed that catalpol can cross BBB into the brain [21] and promote angiogenesis but not aggravate blood-brain barrier leakage in the ischemic brain. Electron microscopic analysis demonstrated that catalpol reduces the edema of microvessels endothelia [22]. Moreover, catalpol can increase the number of synapses [23] and enhance the neuronal axon growth [24], which indicate that catalpol may be a potential protection drug for neurovascular unit. Besides, some researchers reported that catalpol is effective in Alzheimer's disease (AD) [25] and Parkinson's disease (PD) and can improve memory [26]. We also found that stroke rats treated with catalpol are more easily and more quickly to find and grasp the sunflower seeds in the small plate when these rats performed skilled reaching task (data not shown). Evidence above has converged to suggest that catalpol may be a potential agent reducing ischemic damage and enhancing memory. However, the mechanism(s) for these actions has not been well defined.

The present study was aimed at exploring the effects of catalpol on stroke mice and SCOP-induced memory deficits mice, compared with edaravone and oxiracetam. To further study mechanisms of catalpol on stroke and cognitive impairment, ACh, AChE, ChAT, and BDNF in central cholinergic system in hippocampus were investigated. 

## 2. Material and Methods 

### 2.1. Reagent and Drugs

Catalpol was purchased from Liubobainiao Biotechnology Co., Ltd. (Shijiazhuang, China). The purity of the compound was 99% as assayed by high-performance liquid chromatography analysis. ACh and BDNF (brain-derived neurotrophic factor) ELISA kits (R&D system) were purchased from Beijing Dingguochangsheng Biotechnology Co., Ltd. (Beijing, China). TTC (Sigma), Oxiracetam (Guangdong Sencee Pharmaceutical Co., Ltd.), edaravone (Jilin Province Huinan Changlong Bio-pharmacy Co., Ltd.), and SCOP (Hainan Shuangcheng Pharmaceutical Co., Ltd.) were dissolved in 0.9% physiological saline.

### 2.2. Animals

120 Kunming mice were purchased from the Animal Centre, Chongqing Medical University. They (equal numbers of males and females, weighing 25~30 g) were allowed access to water and food ad libitum and maintained at constant temperature (25 ± 1°C) and humidity (55 ± 5%) under a 12 h light/dark cycle (07:00 on to 19:00 off).

### 2.3. The pMCAO Model and Drug Administration

Except for sham group, all groups established a model of permanent middle cerebral artery occlusion (pMCAO) with introducing a suture into the left internal carotid artery (ICA) through the external left carotid artery (ECA) and occluding the middle cerebral artery (MCA). Sham (0.9% saline), model (0.9% saline), edaravone (7.9 mg/kg), oxiracetam (105 mg/kg), and catalpol (9 mg/kg) were administered intraperitoneally 24 h after stroke and then daily for 3 days. 

For the Morris water maze test and biochemical analysis, mice were divided into 7 groups: normal control (normal + 0.9% saline), model (SCOP + 0.9% saline), edaravone (7.9 mg/kg + SCOP) and oxiracetam (105 mg/kg + SCOP) as two positive control groups, three doses of catalpol (1, 3 and 9 mg/kg + SCOP, resp.) as treatment group. Drugs were injected intraperitoneally for three days. In all groups, except for the normal control, learning and memory dysfunction in mice was induced by SCOP (2 mg/kg) intraperitoneal injection 30 min before behavioral testing.

### 2.4. Zea Longa's Score

After operation, the neurological function of all animals was evaluated daily with a 5-point scale as previously described [27]: (0) no neurologic deficit, (1) failure to extend right forepaw fully, (2) circling to the right, (3) falling to the right, and (4) unable to walk spontaneously and had a depressed level of consciousness.

### 2.5. Cerebral Blood Flow Ratios

Three days later, blood flow ratio was measured using a laser Doppler blood flow imager (FLPI, Moor Instruments). The ratio was calculated using the following formula:

(1) $$\begin{array}{c} \text{Blood flow ratios}=\frac{\left[\text{normal }\left(\text{right}\right)-\text{ ischemic }\left(\text{left}\right)\right]}{\text{normal }\left(\text{right}\right)\text{brain}}. \end{array}$$

### 2.6. TTC Staining

Five mice were taken from each of groups to have a TTC staining 3 days after treatment. The brains were taken and cut into coronal sections. Four sections in each brain were obtained and then put in 0.5% TTC PBS solution for 10 min at 37°C without light exposure and then in 4% paraform PBS to fix. The normal brain tissue stained red and infarction area stained white. BI2000 medical image analysis system was used to count the infarction area after the picture was taken.

### 2.7. Morris Water Maze Test

The behavioural procedure of Morris water maze was the same as previously described [28, 29]. The maze was a circular pool (80 cm in diameter and 30 cm in height) filled with water and a nontoxic water-soluble black ink. The black platform (10 cm in diameter and 28 cm in height) was centered in one of the four quadrants of the pool and placed 2 cm beneath the surface of the water. Water temperature was maintained at 25 ± 1°C. The numbers of platform-crossing and escape latencies to find the platform were recorded with a video tracking system. During the test, mice were given four trials per day for 3 days with an intertrial interval 2 min. Once the mice reached the platform, it was permitted to remain on it for 10 s. If mouse failed to reach the platform within 120 s, the escape latencies regarded as 120 s and mice were placed on the platform for 10 s to be induced learning. On the fourth day, the mice were placed in the water at a random point with the platform removed, and the numbers of crossing platform areas were recorded over 120 s. Thirty minutes before the test, mice were injected with SCOP (2 mg/kg) or saline intraperitoneally.

### 2.8. Biochemical Analysis

Following the Morris water maze test, brains were removed after decapitation. The hippocampi were dissected and homogenized containing 10 volumes of cold physiologic saline. The homogenate (10%) was centrifuged at 4000 ×g for 10 min at 4°C. The contents of ACh and BDNF in the hippocampus were measured by ELISA.

### 2.9. Western Blot Analysis

After transcardially perfused with 0.9% NaCl solution to rinse out the blood, mice's hippocampus (0.1 g per brain) in each group was separated and weighed for detecting M_1_, M_2_, AChE, and ChAT by western blot. According to our past reference [22], hippocampus was lysed on ice in lysis buffer (50 mm Tris-HCl (pH 8.2), 0.5 M saccharose, 10 mM HEPES (pH 7.9), 1.5 mM MgCl_2_, 10 mM KCl, 1 mM EDTA, 10% (v/v) glycerine, 1 mM DTT, 1 mM PMSF, 10 *μ*g/mL Aprotinin, and 5 *μ*g/mL Leupeptin), after centrifugation at 12,000 rpm for 5 minutes. Protein content in cleared lysate was determined by Bradford Assay. Lysate samples containing 40 *μ*g of protein were fractionated by SDS-5% polyacrylamide gel electrophoresis and then electroblotted onto PVDF membranes (Millipore, IPVH00010). The membranes were probed with primary antibodies as rabbit anti-mouse- M_1_ (1 : 500, Santa, sc-9106), M_2_ (1 : 2000, Abcam, ab109226), AChE (1 : 500, Santa, sc-11409) and ChAT (1 : 500, Millipore, AB143) polyclonal antibody, and mice anti-rabbit *β*-actin (1 : 1000, Santa, SC-1616R) and then incubated with the horseradish peroxidase-conjugated goat anti-rabbit IgG (1 : 3000; KPL, 074-1506); the PVDF membrane was put into ECL solution. Immunoreactivity was digitally scanned by ScanMaker E6 system and quantified using Alpha Imager Mini (Alpha, American) software. *β*-actin was used as an internal control for all western blotting.

### 2.10. Statistical Analysis

Data were expressed as mean ± SEM All data were analyzed by two-way analysis of variance (ANOVA) using the SPSS 11.5 software. Statistical significance was set at *P* < 0.05.

## 3. Results

### 3.1. Catalpol but Not Oxiracetam Improves Functional Outcome after pMCAO by Zea Longa's Score

**Figure 1:**
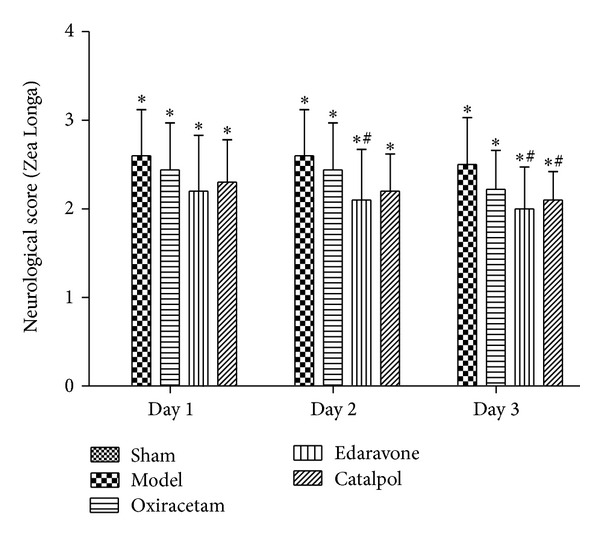
Effect of catalpol on Zea Longa's score at days 1, 2, and 3. The data reported as mean ± SEM (*n* = 10). *P < 0.01 versus sham group. ^#^ *P* < 0.01 versus model group.

Zea Longa's score of the model group was significantly higher than that of the sham group on days 1, 2, and 3 (Figure 1, *P* < 0.01). Compared to the model group, the edaravone group had significantly decreased Zea Longa's score on days 2 and 3 (Figure 1, *P* < 0.01). The catalpol (9 mg/kg) group had significantly decreased score on day 3 (Figure 1, *P* < 0.01). There was no group difference between the oxiracetam and model groups.

### 3.2. Catalpol but Not Oxiracetam Increases the Cerebral Blood Flow

**Figure 2:**
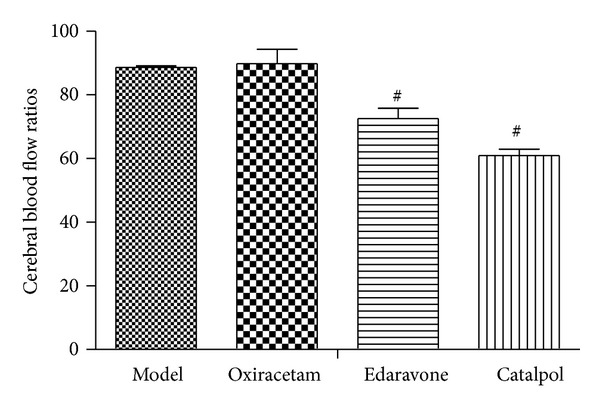
Effect of catalpol on the cerebral blood flow ratios at day 3. The data reported as mean ± SEM (*n* = 10). ^#^ *P* < 0.05 versus model group.

Compared to the model group, the edaravone and catalpol groups significantly decreased cerebral blood flow ratios and increased cerebral blood flow in infarct brain (Figure 2, *P* < 0.05). There was no difference between oxiracetam and model group.

### 3.3. Catalpol but Not Oxiracetam Reduces Infarct Volume after pMCAO

**Figure 3:**
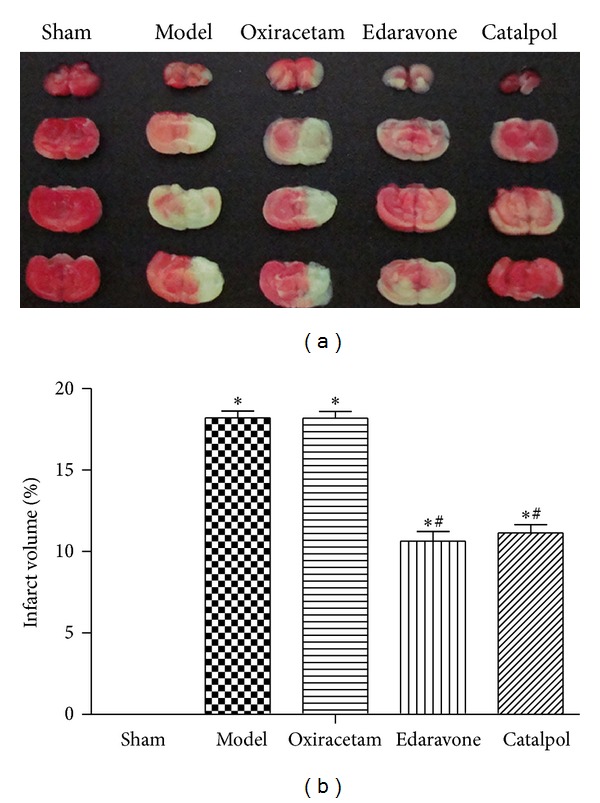
Effect of catalpol on TTC-stained mice brain slice (a) and the infarct volume (b). The data reported as mean ± SEM (*n* = 5). *P < 0.01 versus sham group. ^#^ *P* < 0.05 versus model group.

Treatments with catalpol at 9 mg/kg reduced the total infarct volumes in the permanent model of stroke significantly (Figure 3, *P* < 0.05), and edaravone 7.9 mg/kg also diminished brain damage significantly (Figure 3, *P* < 0.05). However, compared with model group, oxiracetam 105 mg/kg did not reduce the lesion volume in stroke animals (Figure 3, *P* > 0.05). 

### 3.4. Catalpol but Not Edaravone Attenuates the Memory Impairments Induced by Scopolamine in the Morris Water Maze Test

**Figure 4:**
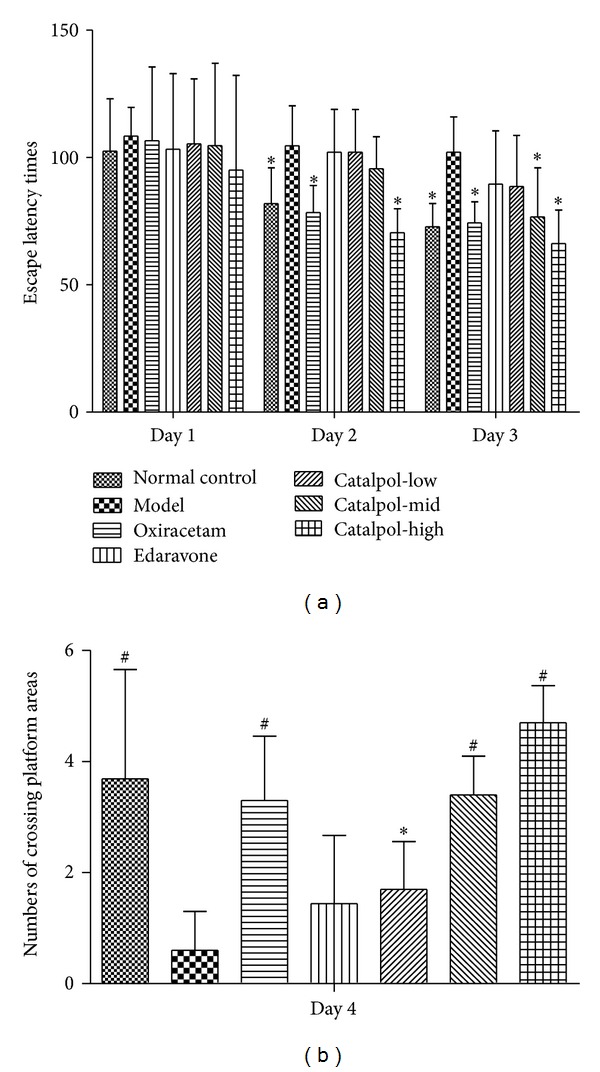
Effect of catalpol on escape latency time (a) and the numbers of crossing platform areas (b). The mice were treated with scopolamine (2 mg/kg i.p.) 30 mins before Morris water maze except for the normal control group. The data represent mean ± SEM (*n* = 10). *P < 0.05 versus model group. ^#^ *P* < 0.01 versus model group.

The effect of catalpol on spatial learning and memory was investigated in the Morris water maze test (shown in Figure 4). The normal control group rapidly learned the location of the platform. The escape latency of the model group was significantly longer than that of the normal control group on days 2 and 3 (Figure 4(a), *P* < 0.05). As compared with the model group, the oxiracetam and catalpol (9 mg/kg) group significantly decreased the escape latency on days 2 and 3 (Figure 4(a), *P* < 0.05) and the catalpol (3 mg/kg) group also significantly decreased it on day 3 (Figure 4(a), *P* < 0.05). Oxiracetam group and catalpol (1, 3 or 9 mg/kg) significantly increased the numbers of crossing platform areas (Figure 4(b), *P* < 0.05). There was no difference between model group and edaravone group (Figure 4(b), *P* > 0.05).

### 3.5. Catalpol but Not Edaravone Increases ACh and BDNF Contents in the Hippocampus

**Figure 5:**
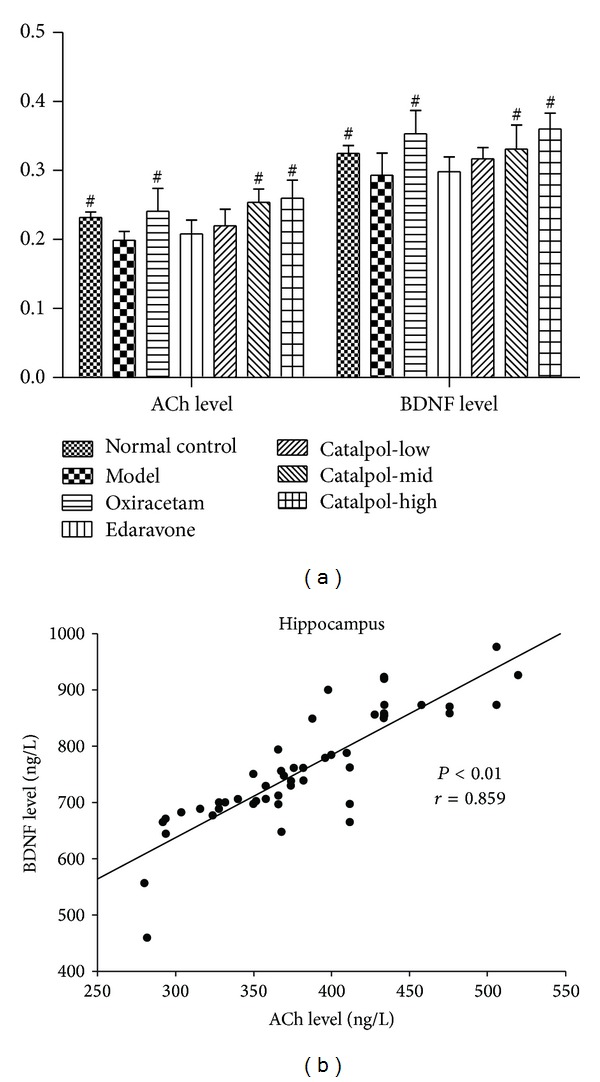
Effect of catalpol on the ACh level (a) and the BDNF level (b), correlations between the ACh level and the BDNF level (b) in hippocampus of mice. The data represent mean ± S.E.M. (*n* = 10). *P < 0.05 versus model group. ^#^ *P* < 0.01 versus model group.

As compared with model group (0.199 ± 0.013 ng/L), the ACh level in the hippocampus significantly increased in the normal control group (0.232 ± 0.008 ng/L), the catalpol (3 or 9 mg/kg, 0.254 ± 0.019 or 0.260 ± 0.026 ng/L), and the oxiracetam groups (0.241 ± 0.033 ng/L) (Figure 5(a), *P* < 0.01). But edaravone group was only 0.208 ± 0.020 ng/L; there was no significant difference with model group (*P* > 0.05). The level of BDNF in the hippocampus of the normal control group (0.325 ± 0.011 ng/L) was significantly higher as compared with the model group (0.293 ± 0.032 ng/L) (Figure 5(a), *P* < 0.05). Oxiracetam (0.353 ± 0.034 ng/L) and catalpol (3 or 9 mg/kg, 0.331 ± 0.035 or 0.360 ± 0.023) significantly increased in the BDNF level (Figure 5(a), *P* < 0.01 versus model group). But BDNF level in edaravone group was only 0.298 ± 0.022 ng/L (*P* > 0.05 versus model). Furthermore, the level of BDNF was positively correlated with the ACh level in the hippocampus (Figure 5(b); *r* = 0.859, *P* < 0.01). 

### 3.6. Catalpol Upregulates ChAT Expression and the Ach Receptor M_1_, M_2_ Level but Reduces AchE Expression in Hippocampus

**Figure 6:**
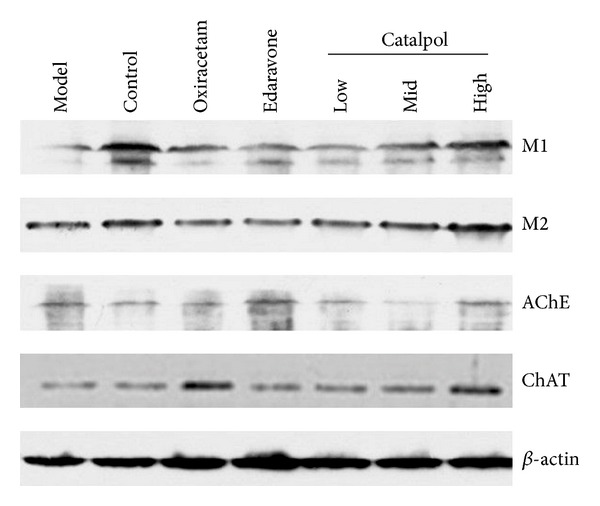
The expression of receptor M_1_, M_2_, AChE, and ChAT in hippocampus (western blot *n* = 5).

Catalpol at 1, 3, 9 mg/kg increased M_1_, M_2_ expression in a dose-dependent manner. Compared with normal group, the Ach receptor M_1_, M_2_ level in the model hippocampus was significantly decreased (Figure 6, *P* < 0.01). This effect was reversed by catalpol and oxiracetam (Figure 6, *P* < 0.01).

As for AChE, although catalpol showed bidirectional regulation on AChE expression, each dose of catalpol decreased AChE expression significantly in hippocampus of mice (Figure 6, *P* < 0.01). Compared with the model group, catalpol at dose of 1, 3 mg/kg reduced AChE expression significantly in a dose-dependent way (Figure 6, *P* < 0.01), but compared with catalpol at dose of 1, 3 mg/kg, catalpol at dose of 9 mg/kg increased the AChE expression, nearing the level of normal group. 

But for ChAT, Catalpol significantly upregulated ChAT level at doses of 1, 3, 9 and mg/kg (Figure 6, *P* < 0.01), respectively. Oxiracetam also significantly reduced AChE and upregulated ChAT levels. However, edaravone increased both AChE and ChAT significantly (Figure 6, *P* < 0.01). 

**Figure 7:**
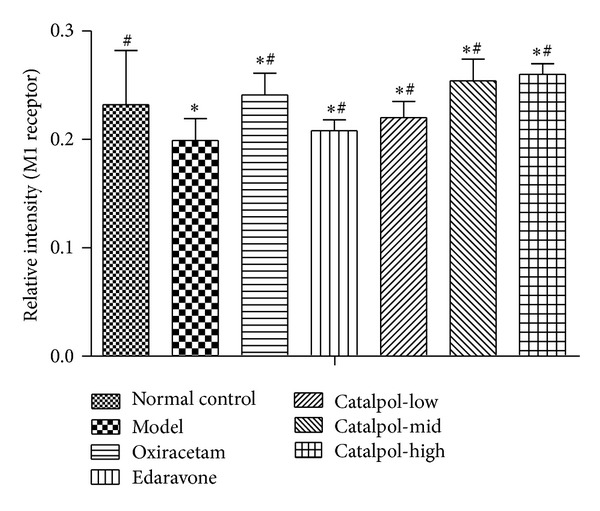
Western blot analysis of the effect of catalpol on the expression of M_1_ in hippocampus (*x* ± *s*, *n* = 5). *P < 0.01 versus normal group. ^#^ *P* < 0.01 versus the model group.

**Figure 8:**
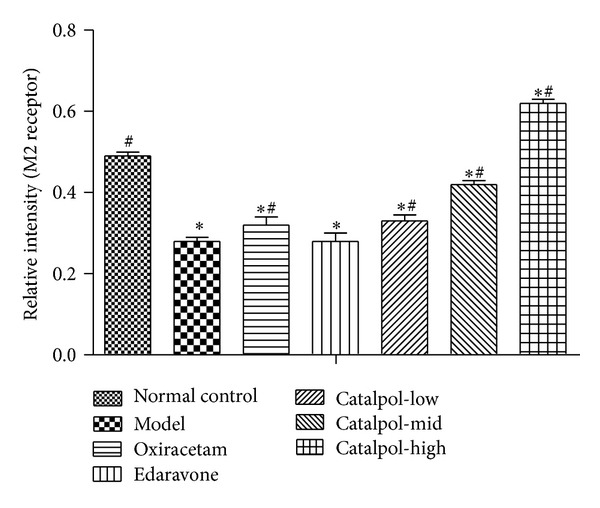
Western blot analysis of the effect of Catalpol on the expression of M_2_ in hippocampus (*x* ± *s*, *n* = 5). *P < 0.01 versus normal group. ^#^ *P* < 0.01 versus model group.

**Figure 9:**
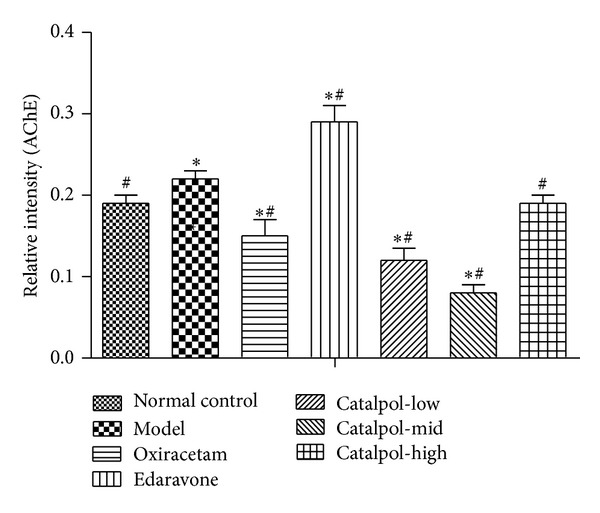
Western blot analysis of the effect of Catalpol on the expression of AChE in hippocampus (*x* ± *s*, *n* = 5). *P < 0.01 versus normal group. ^#^ *P* < 0.01 versus model group.

**Figure 10:**
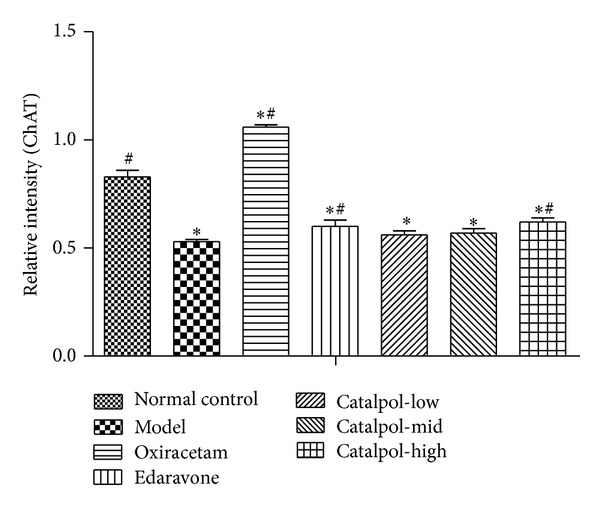
Western blot analysis of the effect of Catalpol on the expression of ChAT in hippocampus (*x* ± *s*, *n* = 5). *P < 0.01 versus normal group. ^#^ *P* < 0.01 versus model group.

The statistical graph of expression of receptor M_1_, M_2_, AChE, and ChAT in hippocampus is shown in Figures 7, 8, 9, and 10, respectively.

## 4. Discussion

Cognitive impairment is a common consequence of stroke and impacts on recovery of sensorimotor functions after stroke [8]. However, there is a lack of drugs that not only improve cognitive status effectively but also promote function recovery after stroke. The dilemma has raised the discovery of new drugs to an issue of major importance. 

The present work revealed that catalpol has a neuroprotective effect against cerebral ischemic damage and memory-enhancing effects and disclosed a novel mechanism; that is, catalpol could improve memory function via mediating Cholinergic signaling pathway. 

In our study, we employed cerebral ischemic stroke model to confirm catalpol's neuroprotective effects. As expected, an obvious improvement of neurological function was found in ischemic mice treatment with catalpol, a marked reduction in the infarction volume validated the catalpol's neuroprotection against cerebral ischemic damage. Further studies demonstrated that catalpol facilitated cerebral blood flow restoration, which would be in accordance with infarct volume reduction. However, oxiracetam, a nootropic agent, which is known to improve both learning and memory processes and used for treatment of various cognitive disorders, did not reduce the infarction volume. The experimental results presented here, in line with previous observations [30, 31], supported that catalpol may be a promising candidate as a treatment of choice for neuroprotection after stroke.

Scopolamine (SCOP), a blocker of muscarinic acetylcholine receptor (mAChR), induces cognitive deficit in various animals [32]. Acute and systemic administration of SCOP in young animals provides the appropriate memory deficits related to the cholinergic deficit in AD, senile CNS dysfunction, or poststroke cognitive impairment. So SCOP-induced amnesic model has been widely used to provide a pharmacological model of memory dysfunction for screening potential cognition enhancing agents [33, 34]. In the present study, the effect of improving memory deficit of catalpol was evaluated using the amnesic mouse model induced by SCOP using Morris water maze test and biochemical assessments. In the Morris water maze test, if the animals spent more time and swam a longer distance in the pool quadrant where the platform had previously been placed during the training session, this would indicate that the animals learned from prior experience with the Morris water maze test, showing the spatial memory improvement [31, 35]. Mice treated with SCOP showed a more prolonged escape latency than mice in the normal control group. Catalpol and oxiracetam, but not edaravone, lowered the escape latency and significantly increased the number of times of crossing over the platform site comparable to the control group. It is important to notice that the Morris water maze test investigating spatial learning and memory has been used in detecting changes of the central cholinergic system [33, 36]. Therefore, these results suggested that catalpol can improve the long-term memory in SCOP-induced memory impairments and may be involved in mediation of the cholinergic nervous system.

Previous studies suggested that the central cholinergic system plays an important role in learning and memory [37]. Damage to the cholinergic system (acetylcholine producing) in the brain has been shown to be plausibly associated with the memory deficits associated with Alzheimer's disease [38] and poststroke dementia (PSD) [17, 18]. After release from the nerve terminal, ACh may bind with cholinoceptors or split into choline and acetate by acetylcholinesterase (AChE). In an in vivo study as a model of dementia treated with SCOP, cholinergic neurotransmission was obstructed leading to an increase of the AChE and impaired cognition [19]. The cholinesterase inhibitors act primarily where ACh is released and work as an amplifier of endogenous ACh. ChAT, the biosynthetic enzyme for ACh, is presently the most specific cholinergic marker for checking the functional state of cholinergic neurons in the CNS and peripheral nervous system [39, 40]. According to the cholinergic hypothesis, memory impairments in patients with senile dementia are due to a selective and irreversible deficiency in the cholinergic functions in the brain [41, 42]. Therefore, downregulation of AChE and upregulation of ChAT may compensate for reduced ACh levels in brains with AD disease or stroke and may facilitate ischemia-induced memory functional recovery. 

To elucidate the underlying mechanisms of memory enhancing effects of catalpol, contents of ACh, AChE, ChAT, and muscarinic ACh receptors M_1_ and M_2_ as cholinergic markers were assessed using hippocampus homogenates. The current work displayed that catalpol and oxiracetam both significantly increased the ACh concentration in the hippocampus. Further examinations demonstrated that catalpol treatment mainly upregulated the expression of ChAT in a dose-dependent manner but did not show dose dependently downregulated expression of AChE in hippocampus. Although each dose of catalpol significantly reduced AChE expression compared with the model group, middle (3 mg/kg) dose of catalpol induced the lowest expression level in AChE compared to the high (9 mg/kg) dose, which may be a negative feedback from the higher dose. This phenomenon may be in part explained by that high concentration of neurotransmitters such as acetylcholine or glutamate would suppress the synaptic transmission by an action at a presynaptic autoreceptor and activate its degrading enzyme production such as AChE, which results in a U-shaped dose-response curve [43]. Further studies would be needed to explore the interesting dosage effects and its exact mechanism(s). Oxiracetam treatment not only significantly reduced AChE but also increased ChAT. However, edaravone obviously increased both AChE and ChAT. It is well known that AChE accelerates ACh degradation, which neutralizes ChAT action on ACh production, and this finding well explained why edaravone fails to improve learning or memory behaviour in our study. In fact, partly in consistent with our result, scopolamine caused impairment in memory associated with reduced acetylcholine (ACh) level and elevated acetylcholinesterase (AChE) activity previously [44]. Oxiracetam (100 mg/kg, ip) significantly prevented the SCOP-induced memory impairment in mice [14], and edaravone (6 mg/kg) gave no protection to the learning and memory capability in a rat model of neonatal hypoxic-ischemic encephalopathy; 9 mg/kg edaravone has no amelioration on learning and memory deficit at 5 d or 10 d [45]. Doses of Oxiracetam and edaravone were chosen in our study on the basis of previously conducted behavioral studies that produced differential effects in cognitive performance, when the body surface area normalization method was used. Thus, modification of cholinergic systems via modulation of AChE, ChAT protein expressions, and elevation of ACh level was, at least in part, linked closely with the antiamnesic effect of catalpol on SCOP-induced impairment of learning and memory. 

Muscarinic acetylcholine receptors (mAChR) control the time course of evoked ACh release [46] and play essential role in memory formation. Until now, at least five well-characterized subtypes of mAChR (M_1_–M_5_) have been found in the brain, in which M_1_ and M_2_ are mainly expressed in the hippocampus and cortex [47, 48]. M_1_ has a close relation with motor and memory; M_2_ has a relation to ACh release; antagonism of M_2_ will worsen memory impairment [48]. Our results showed that SCOP-induced mice had lower level of M_1_ and M_2_ in the hippocampus than mice from the normal control group. Catalpol and oxiracetam reversed the effects partly. Notably, Catalpol upregulated the M_1_ and M_2_ expression in a dose-dependent manner, and even more, the M_2_ level in catalpol groups (except for 1 mg/kg) was significantly higher than that of the normal control group. Thus, it could be deduced that catalpol-induced cognitive ameliorative effects may be related to controlling ACh release via regulating the expression of muscarinic ACh receptors, which implies that catalpol has multiple action sites in the cholinergic pathway.

Brain-derived neurotrophic factor (BDNF) is a key regulator in the formation of memory [49], and there are positive feedbacks between ACh and BDNF in the rat hippocampus [50]. BDNF interaction with tropomyosin-related kinase B receptors and presynaptic muscarinic receptors modulates transmitter release in adult rodent motor nerve terminal, which can improve stroke motor function recovery [51]. Previous studies reported that catalpol increased the hippocampal neuroplasticity in the aged rats [52] and attenuated MPTP-induced neuronal degeneration of nigral-striatal dopaminergic pathway in mice [53] partly attributed to BDNF upregulation. At the same time, higher BDNF level, together with ACh, is beneficial to brain plasticity and the induction of specific, associative detail behavioral memory [54, 55] and synaptogenesis [56]. In line with previous studies, our results showed that catalpol can upregulate BDNF level and here existed the strong positive correlation between the ACh level and the BDNF level in the hippocampus (*r* = 0.859, *P* < 0.01), which are together beneficial to memory improvement and to explain from another side why catalpol ameliorated memory impairments in the SCOP-induced amnesic mice. 

In summary, we report in this experiment that advantage of catalpol is its ability not only to protect ischemic damage but also improve memory through multiple mechanisms of action, including increasing ACh production by the promotion of ChAT, inhibition of AChE, and upregulating M_1_ and M_2_ expression together with increasing BDNF generation. The development of such herbal medicines capable of targeting multiple sites could be useful for future drug discovery and the potential management of stroke diseases.
